# Association between Anti-Helicobacter pylori IgG seropositivity on coronary heart disease and potential pathogenesis: a Mendelian randomization study

**DOI:** 10.1371/journal.pone.0329137

**Published:** 2025-12-09

**Authors:** Jing Wang, Tianying Chang, Yiqiang Wang, Jinling Zhang, Rui Chen, Le Liu, Yingzi Cui, Hongguang Jin

**Affiliations:** 1 Changchun University of Chinese Medicine, Changchun, China; 2 EBM office, the Affiliated Hospital to Changchun University of Chinese Medicine, Changchun, China,; 3 Department of Cardiology, the Affiliated Hospital to Changchun University of Chinese Medicine, Changchun, China; Cardiff University, UNITED KINGDOM OF GREAT BRITAIN AND NORTHERN IRELAND

## Abstract

**Background:**

Currently, observational studies and clinical trials have shown that the occurrence of coronary heart disease (CHD) is closely related to anti-Helicobacter pylori (H. pylori) IgG seropositivity, but these studies may be affected by confounding factors, resulting in a causal relationship that is still controversial.

**Methods:**

We conducted a Mendelian randomization analysis to clarify the association between anti-H. pylori IgG seropositivity and CHD and explore its potential pathogenesis. Exposure, outcome, and mediator data were obtained all from the genome-wide association studies (GWAS) summary data. The inverse variance weighting (IVW) analysis method under the fixed-effect model was used as our primary statistical method. Other statistical methods are supplements, such as IVW under the random-effects model, weighted median, and MR-Egger regression method. Causal estimates are presented as odds ratios (ORs) with 95% confidence intervals (CIs). To ensure the stability and reliability of the results, we also performed sensitivity and heterogeneity analysis.

**Results:**

This study shows that increases of anti-H. pylori IgG seropositivity was significantly associated with an increased risk of CHD (OR=1.003, 95% CI:1.0001.006, P = 0.048) and increases in peak insulin response (β = 0.214, 95% CI: 0.025–0.403, P = 0.026), decrease in total free cholesterol levels (β = ‒0.031, 95% CI: ‒0.061 to ‒0.000, P = 0.045), decreased waist circumference (β = ‒0.073, 95% CI: ‒0.138 to ‒0.008, P = 0.027) and waist-to-hip ratio (β = ‒0.069, 95% CI: ‒0.136 to ‒0.002, P = 0.044). The leave-one-out method proved inadequate in detecting instances where a single SNP exhibited a bias towards or dependency on the causation. Furthermore, our findings revealed no heterogeneity or pleiotropy.

**Conclusion:**

Our MR study provided strong evidence of a causal relationship between anti-H. pylori IgG seropositivity and related to an increased risk of CHD. The effect of anti-H. pylori IgG seropositivity on CHD may be related to a higher peak insulin response or lower total free cholesterol levels and a decrease in waist circumference and waist-to-hip ratio. However, further research is needed to prove the results.

## 1. Introduction

As the incidence and prevalence of coronary heart disease (CHD) are increasing every year, it is placing a heavy burden on the global economy [1,2]. According to the Global Burden of Illness study, cardiovascular disease was the underlying cause of 9.6 million deaths among men and 8.9 million deaths among women, approximately one-third of all deaths globally [3]. Due to the high morbidity and mortality, it is essential to explore novel risk factors of CHD and carry out early prevention and intervention [4]. A variety of potential risk factors cause CHD. Modifiable risk factors for CHD include dyslipidemia, hypertension, diabetes, smoking, obesity, body mass index, waist circumference, etc [5]. Reduced modifiable risk factors can improve CHD prevention and control, which is essential for the early prevention of CHD [6]. Evidence suggests that inflammation is a critical factor in the development and progression of CHD [7]. Chronic infection with various pathogens may trigger inflammatory responses in blood vessel walls, which may be crucial in developing atherosclerosis and progressing CHD [8–10].

Helicobacter pylori (H. pylori) is a gram-negative bacterium that infects more than half of the world’s population [11,12]. The transmission of H. pylori is mainly carried out through oral⁃oral and fecal⁃oral routes. H. pylori infection is a significant health burden, and although a large proportion of infections are asymptomatic or exhibit relatively mild non-specific symptoms, infection with this pathogen may lead to gastroduodenal pathology and is strongly associated with the occurrence and development of some extra parenteral diseases. H. pylori infection involves multiple systems [13]. A large number of observational studies have indicated that H. pylori infection is concerned with CHD and uro-gynecologic diseases, as well as diabetes mellitus and metabolic syndrome [14–17]. H. pylori infection is a risk enhancer factor for atherosclerotic cardiovascular diseases [18]. In recent years, a substantial number of clinical studies have confirmed connections between H. pylori IgG seropositivity for CHD [19–22]. However, these studies mostly use traditional research methods, which confounding factors may influence. These unknown and residual confounding factors may be affecting the results. Therefore, whether H. pylori plays a causal role in the CHD remains unclear. It is urgent to further clarify the relationship between anti-H. pylori IgG seropositivity and CHD and explore its pathogenesis. The underlying mechanisms between anti-H. pylori IgG seropositivity and CHD remain undiscerned. In recent decades, epidemiologic and clinical trials addressing connections between H. pylori infection and risk factors for CHD have been revealed. Firstly, H. pylori infection and CHD are related to inflammation accompanied by C-reactive protein, tumor necrosis factor alfa, and homocysteine [23]. Secondly, clinical studies have shown that obesity is strongly associated with the elevated risk of CHD [24]. Obesity is defined by body mass index (BMI). However, for any given BMI, body fat distribution can vary substantially. Waist circumference is an important indicator of abdominal adiposity. In addition, the waist-to-hip ratio is also a measure of abdominal adiposity. Furthermore, a study concluded that H. pylori infection is as risk factor for the development of insulin resistance and cardiovascular disease [25]. Finally, blood pressure and lipids are two important risk factors for CHD. Several cross-sectional studies have also shown that H. pylori infection is associated with hypertension and dyslipidemia [17,26].

As a popular method of genetic epidemiology study, Mendelian randomization (MR) explores the causal link between exposures and outcomes via genetic variants [27]. MR research uses single nucleotide polymorphisms (SNPs) as instrumental variables to infer and evaluate causality [28]. Due to genetic variants being randomly assigned during the formation of human gametes, and genotypes were typically unaffected by the external environment, the MR method could largely avoid and overcome the limitations of confounding factors and reverse causality common in observational studies [29,30]. In addition, genome-wide association studies (GWAS) have accumulated many associations between genetic variants and phenotypes. Therefore, with sufficient sample size and without additional ethical approval, these data can be used to assess the causal relationship between exposure and outcomes effectively. Our literature review found that previous studies had confirmed a causal relationship between anti-H. pylori IgG seropositivity and cardiovascular disease [31]. The study used a two-sample MR analysis to clarify their causal relationship, explore its potential pathogenesis, and provide helpful advice for clinical early prevention of CHD.

## 2. Methods

### 2.1. Study design

**Fig 1:**
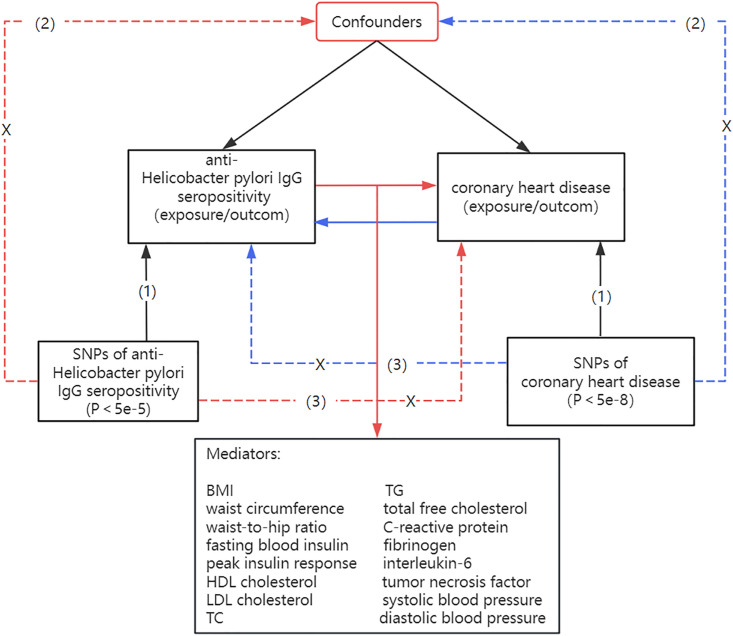
Three essential assumptions of the Mendelian randomization study. (SNPs, single-nucleotide polymorphisms; BMI, body mass index; LDL, low density lipoprotein; HDL, high density lipoprotein; TG, triglycerides; TC, total cholesterol; H. pylori, Helicobacter pylori. [1] The genetic variants are robustly associated with exposure; [2] The genetic variants are not associated with confounders; [3] The genetic variants can only induce outcomes through exposure).

We applied an MR design to elucidate the causal association between anti-H. pylori IgG seropositivity on CHD and mediators. An MR research design must be based on the following three core assumptions:(1) the instrumental variables (IVs) are significantly associated with anti-H. pylori IgG seropositivity; (2) the IVs should not be related to potential confounding factors that affect both exposure or outcome; (3) the IVs should exert effects on the outcome only through the exposure (Fig 1).

### 2.2. Data source and selection of SNPs

We obtained the publicly available GWAS summary statistic for anti-H. pylori IgG seropositivity, which included 8,735 European samples and number of 9,170,312 SNPs [32]. We used summary statistics data sets of IEU Open GWAS data for CHD. This CHD study data included 361,194 cases and 351,037 controls, all of European ancestry. It contains 13,295,130 SNPs. Possible pathogenesis underlying the association between H. pylori and CHD provides blood pressure, fasting blood insulin, peak insulin response, BMI, Waist circumference, waist-to-hip ratio, dyslipidemia, and inflammation factors. Dyslipidemia includes low-density-lipoprotein cholesterol (LDL), high-density lipoprotein cholesterol (HDL), triglycerides (TG), total cholesterol (TC), and free cholesterol. Blood pressure, including diastolic blood pressure and systolic blood pressure. Inflammatory factors include interleukin, tumor necrosis, C-reactive protein, and fibrinogen. These mediator data are from IEU Open GWAS (Table 1). Data were obtained from four ethnic populations, including LDL cholesterol and systolic blood pressure from East Asian; HDL-C, TC and TG from Sub-Saharan African; peak insulin response and fasting blood insulin from Hispanic or Latin American. The rest of the data are from European. Among them, the data on waist circumference and waist-to-hip ratio are from the European female population.

**Table 1:** Details of the GWAS data in this study.

Phenotype	GWAS ID	Year	Population	Sample size	Number of SNPs
Anti-H. pylori IgG seropositivity	ebi-a-GCST90006910	2020	European	8,735	9,170,312
coronary heart disease	ukb-d-I9_CHD	2018	European	361,194	13,295,130
BMI	ebi-a-GCST90025994	2021	European	457,756	4,238,669
Waist circumference	ieu-a-69	2015	European	127,469	2,473,036
Waist-to-hip ratio	ieu-a-81	2015	European	116,740	2,467,779
LDL-C	bbj-a-31	2019	East Asian	72,866	6,108,953
HDL-C	ebi-a-GCST90101746	2022	Sub-Saharan African	24,616	21,361,416
TC	ebi-a-GCST90101743	2022	Sub-Saharan African	10,486	13,930,870
TG	ebi-a-GCST90101748	2022	Sub-Saharan African	24,600	21,308,171
Total free cholesterol	ebi-a-GCST90092988	2022	European	115,082	11,590,399
Peak insulin response	ebi-a-GCST006676	2017	Hispanic or Latin American	2,337	9,636,653
Fasting blood insulin	ebi-a-GCST008033	2019	Hispanic or Latin American	12,687	28,622,174
Tumor necrosis factor	prot-a-3029	2018	European	3,301	10,534,735
C-reactive protein	ebi-a-GCST90029070	2022	European	575,531	10,713,245
Fibrinogen	ebi-a-GCST005062	2017	European	9,762	17,332,326
Interleukin-6	ebi-a-GCST004446	2016	European	8,189	9,790,590
SBP	ieu-b-5075	2021	East Asian	145,505	13,230,376
DBP	ebi-a-GCST90025981	2021	European	422,713	4,228,468

BMI, body mass index; LDL-C, low-density-lipoprotein cholesterol; HDL-C, high density lipoprotein cholesterol; TC, total cholesterol; TG, triglyceride; SBP, systolic blood pressure; DBP, diastolic blood pressure.

Based on the above data, we performed a two-sample MR analysis. Firstly, when we set the p-value threshold as 5 × 10^−8^, we did not select any independent SNPs associated with anti-H. pylori IgG seropositivity as the IVs. Subsequently, to contain more independent SNPs that are concerned with anti-H. pylori IgG seropositivity, we relaxed the criteria for trial analysis (P < 5 × 10^−5^), which had been applied to previous MR study [33,34]. Under this standard, we selected a total of 84 SNPs that were significantly associated with anti-H. pylori IgG seropositivity. When data for exposure and outcomes were combined, we found 81 SNPs. Secondly, we used the p-value threshold as 5 × 10^−8^ in the reverse MR analyses, in which we discovered 12 SNPs related to CHD. In the study, the clumping process (a linkage disequilibrium (LD) r^2^ < 0.001 and clumping distance = 10,000 kb) was conducted to assess the LD between the included SNPs [35]. In addition, to avoid weak instrument bias, we calculated the F-statistics for each SNP with the equation: F = R^2^×(N − 2)/ (1 − R^2^), R^2 ^= 2×(1 − MAF)×(MAF)×β^2^. There may be the effect of potential bias when the F ＜ 10, the SNPs were regarded as weak IVs [36, 37] and excluded. The results show that the F-statistics varied from 16 to 26, nothing less than 10.

### 2.3. Statistical analysis for MR

MR analysis of genetic variation must be related to exposure but not affected by confounders. Firstly, the SNPs related to the exposure and outcome were standardized to ensure alignment of allelic directions and removal of palindromic sequences. Then, SNPs were excluded if unavailable in outcome datasets or defined as ambiguous palindromic SNPs with minor allele frequencies >0.42 and <0.58. In this study, three SNPs were eliminated. Moreover, before we conducted MR analysis, we used the MR-PRESSO method to detect outliers to enhance the robustness of the results. The statistical power of the inverse-variance weighted (IVW) method is significantly higher than that of other MR methods. It assumes that all genetic variation is valid and statistically most robust when the mean pleiotropic effect is zero [38,39]. No heterogeneity was found in this study. Therefore, we primarily adopted the IVW method under the fixed-effect model in this MR study. In addition, multiple complementary analyses were conducted to ensure the results’ stability and reliability, such as IVW under the random-effects model, weighted median, and MR-Egger. To validate the dependability of the primary findings, conducting sensitivity analysis has been pivotal in uncovering potential pleiotropy and heterogeneity within MR estimates. The MR-Egger regression intercept can indicate the pleiotropic effects of IVs [40]. The MR-Egger slope of this regression represents the causal effect estimate, and the intercept can be interpreted as an estimate of the average horizontal pleiotropic effect across the genetic variants [41]. Pleiotropic bias was presumed to be present if the MR-Egger intercept yielded a significance level of P < 0.05. Horizontal pleiotropy means that IVs for exposure can affect outcomes through multiple independent pathways. Therefore, we utilized the MR-PRESSO method to identify outliers to verify the presence of potential level pleiotropy before proceeding with MR estimation. Moreover, we assessed heterogeneity between SNPs using Cochran’s Q-statistics before performing statistical tests for heterogeneity between SNPs to avoid biased estimates of causal effects [42]. Additionally, sensitivity analyses were performed. To determine whether a single SNP drives the causal signal, we also conducted a Leave-one-out analysis. To explore whether CHD has any causal impact on the identified significant anti-H. pylori IgG seropositivity, we also performed a reverse MR analysis using the same analytical method. A total of MR analyses was performed using the RStudio package“TwosampleMR”. Results are reported following the STROBE-MR (Strengthening the Reporting of Observational Studies in Epidemiology—Mendelian Randomization) statement.

### 2.4. Ethics declaration

No additional ethical review was required since our study used publicly available summary data.

## 3. Results

### 3.1. Causal effects of anti-H. pylori IgG seropositivity on CHD

**Fig 2:**
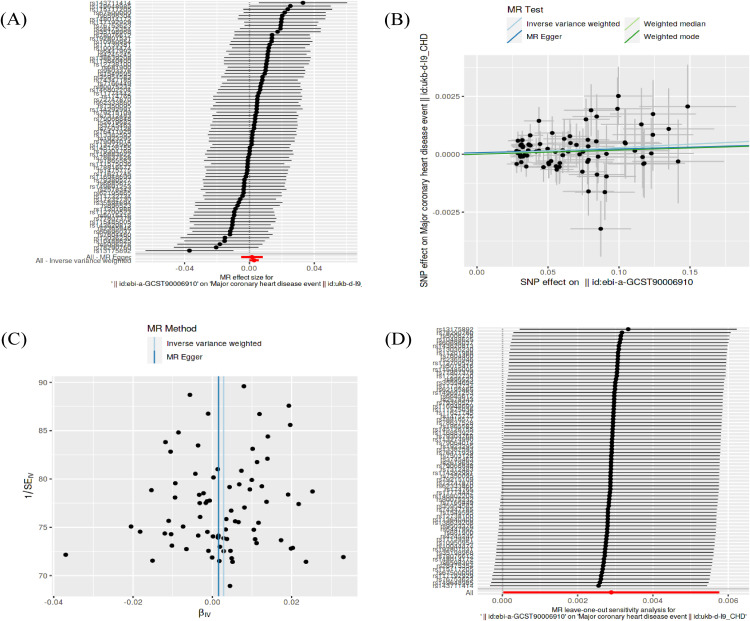
Mendelian randomization analysis of the effect of anti-H. pylori IgG seropositivity on coronary heart disease (CHD). (A) Forest plot showing the odds ratios (ORs) and 95% confidence intervals (CIs) for the causal effect of anti-H. pylori IgG seropositivity on CHD risk using different MR methods. (B) Scatter plot of the genetic associations with the exposure against the genetic associations with the outcome. The slopes of the lines represent the causal estimate from different MR methods. (C) Leave-one-out sensitivity analysis, where each point represents the IVW model estimate after systematically removing the indicated single-nucleotide polymorphism (SNP). (D) Funnel plot for assessing the potential pleiotropy of the instrumental variables. IVW, inverse variance weighted; WM, weighted median.

Before we performed MR estimation, no outliers with large pleiotropy were detected by MR-PRESSO analysis in this study. In this study, three ambiguous palindromic SNPs (rs7281117, rs761648, rs8051818) defined as having minor allele frequencies were eliminated. We ultimately selected 78 SNPs for this study. In this MR analysis, the IVW method analysis under fixed-effect showed evidence to support a causal association between anti-H. pylori IgG seropositivity and CHD (OR=1.003 95% CI:1.0001.006, P = 0.048). Under a random-effects model, we obtained similar risk results (OR=1.003, 95% CI:1.000–1.005, P = 0.026). However, the remaining two analyses showed no causal association between anti-H. pylori IgG seropositivity and CHD. The results of the MR-Egger regression analysis showed no causal association between anti-H. pylori IgG seropositivity and CHD (OR=1.002, 95% CI:0.995–1.008, P = 0.644; Table 2, Fig 2A). The weighted median method did not provide evidence of a causal association between anti-H. pylori IgG seropositivity and CHD (OR=1.003, 95% CI:0.999–1.007, P = 0.172; Table 2, Fig 2A). The association between anti-H. pylori IgG seropositivity and CHD were inconsistent between IVW and the additional two methods. However, the statistical power of the IVW method is significantly higher than that of other MR methods [43]. Furthermore, we performed sensitivity analyses and heterogeneity pleiotropy tests to ensure the reliability and robustness of the results. The MR-PRESSO method found no horizontal pleiotropy after excluding the pleiotropic variant. MR-Egger regression analysis showed no horizontal pleiotropy (intercept = 7e-05, SE = 0.00016, p = 0.671; Fig 2B). Then, to test the heterogeneity of the study, Cochran’s Q test indicated no evidence of heterogeneity between IV estimates based on the individual variants (MR Egger: P = 0.900, IVW: P = 0.912; Fig 2C). We then used the leave-one-out study to test the robustness of the results. Results from the leave-one-out analysis demonstrated that no single SNP was driving the IVW point estimate (Fig 2D). Overall, there is no horizontal pleiotropy and heterogeneity in this study.

**Table 2:** Mendelian randomization analyses for the relevance of anti-H. pylori IgG seropositivity with risk of CHD.

Exposure	Outcome	Method	nsnp	OR (95%CI)	P-value	Heterogeneity		Pleiotropy	
						Q	P	Intercept	P
anti-H. pylori IgG seropositivity	CHD	MR Egger	78	1.002(0.995-1.008)	0.644	60.67	0.900	−0.00059	0.913
		IVW	78	1.003(1-1.006)	0.048	60.85	0.912		
		WM	78	1.003(0.999-1.007)	0.159	—	—		
CHD	anti-H. pylori IgG seropositivity	MR Egger	12	1.121(0.079-15.955)	0.935	4.97	0.893	7e-05	0.671
		IVW	12	0.979(0.290-3.302)	0.973	4.98	0.932		
		WM	12	0.893(0.178-4.471)	0.891	—	—		

CHD, coronary heart disease; nsnp, numbers of single-nucleotide polymorphism; IVW, inverse variance weighted method; WM, weighted median; OR, odds ratio; CI, confidential interval.

### 3.2. Causal effects of anti-H. pylori IgG seropositivity on potential pathogenesis

**Fig 3:**
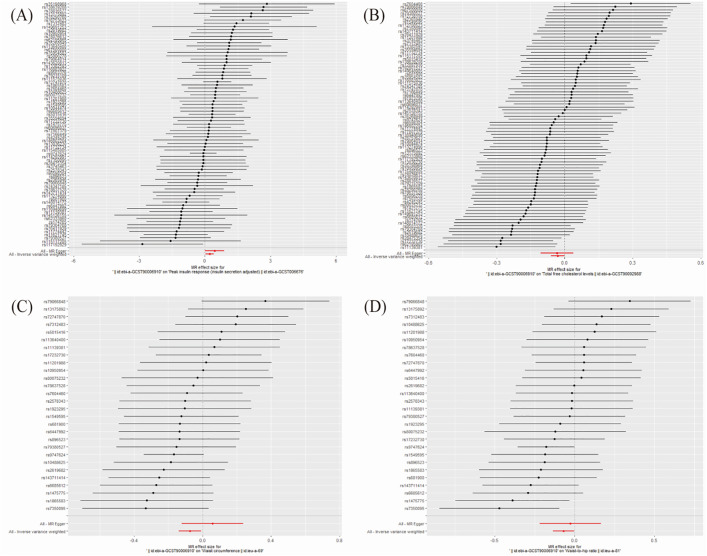
Forest plot for the significant MR association between four mediators and anti-H. pylori IgG seropositivity. (A) Peak insulin response. (B) Total free cholesterol levels. (C) Waist circumference. (D) Waist-to-hip ratio. MR estimates were generated using the inverse-variance weighted method. Beta values represent the change in the mediator per unit increase in the genetic liability to anti-H. pylori IgG seropositivity.

We performed MR-PRESSO analysis to identify outliers exhibiting pleiotropy and conducted MR estimates. In our study, we discovered that anti-H. pylori IgG seropositivity correlates with the increase in peak insulin response (β = 0.214, 95% CI: 0.025–0.403, P = 0.026; Fig 3A) and a decrease in total free cholesterol levels (β = ‒0.031, 95% CI: ‒0.061 to ‒0.000, P = 0.045; Fig 3B). In addition, our study found that anti-H. pylori IgG seropositivity is associated with reduced waist circumference (β = ‒0.073, 95% CI: ‒0.138 to ‒0.008, P = 0.027; Fig 3C) and waist-to-hip ratio (β = ‒0.069, 95% CI: ‒0.136 to ‒0.002, P = 0.044; Fig 3D) in the European female population.

**Fig 4:**
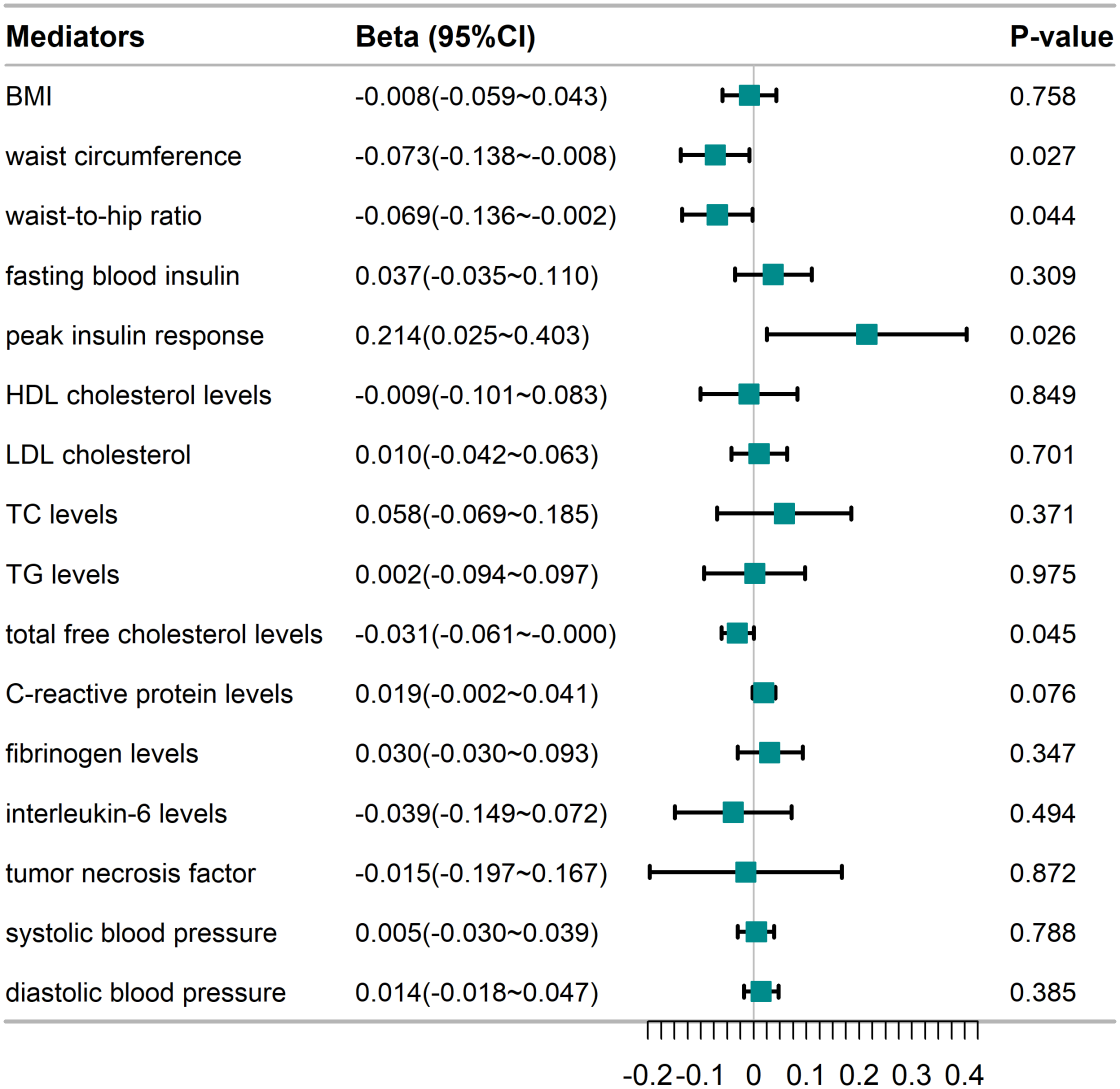
Associations of anti-H. pylori IgG seropositivity with mediators. CI, confidence interval; BMI, body mass index, LDL, low density lipoprotein; HDL, high density lipoprotein; TG, triglyceride. The effect sizes (Beta) and 95% CIs for the causal relationship between anti-H. pylori IgG seropositivity and each potential mediator are shown.

Our study found no evidence of association between anti-H. pylori IgG seropositivity and BMI (β = ‒0.008, 95% CI: ‒0.059–0.043, P = 0.758), LDL cholesterol (β = 0.010, 95% CI: ‒0.042–0.063, P = 0.701), HDL cholesterol levels (β = ‒0.009, 95% CI: ‒0.101–0.083, P = 0.849), TC levels (β = 0.058, 95% CI: ‒0.069–0.185, P = 0.371), TG levels (β = 0.002, 95% CI: ‒0.094–0.097, P = 0.975), fasting blood insulin (β = 0.037, 95% CI: ‒0.035–0.110, P = 0.309), tumor necrosis factor (β = ‒0.015, 95% CI: ‒0.197–0.167, P = 0.872), C-reactive protein levels (β = 0.019, 95% CI: ‒0.002–0.041, P = 0.076), fibrinogen levels (β = 0.030, 95% CI: ‒0.03–0.093, P = 0.347), interleukin-6 levels (β = ‒0.039, 95% CI: ‒0.149–0.072, P = 0.494), systolic blood pressure (β = 0.005, 95% CI: ‒0.030–0.039, P = 0.788), diastolic blood pressure (β = 0.014, 95% CI: ‒0.018–0.047, P = 0.385) (Fig 4).

**Fig 5:**
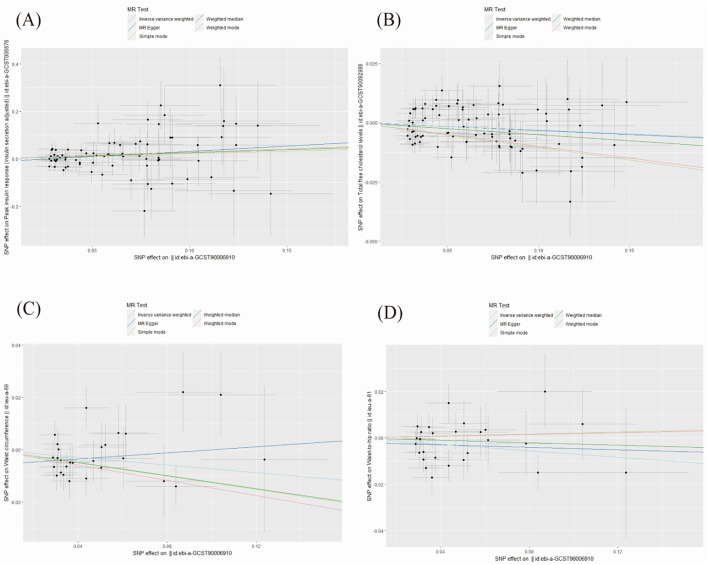
Scatter plot for the significant Mendelian randomization (MR) association (P < 0.05) between mediators and anti-H. pylori IgG seropositivity. Each point represents the association between a single-nucleotide polymorphism (SNP) with the exposure (anti-H. pylori IgG seropositivity) and the outcome (mediator). The slopes of the lines represent the causal estimate from different MR methods.

It was plotted using the statistical software Free Statistics (version 1.9.2). To verify the robustness of the causal relationship between anti-H. pylori IgG seropositivity and the four mediators involved, we performed sensitivity analyses, horizontal pleiotropy and heterogeneity test. The analysis found no heterogeneity and no horizontal pleiotropy (Fig 5) between these four mediators and anti-H. pylori IgG seropositivity.

And our research found that the MR estimates were not driven by a single SNP.

### 3.3. Causal effects of CHD on anti-H. pylori IgG seropositivity

To explore whether CHD has any causal impact on anti-H. pylori IgG seropositivity, we also performed a reverse MR analysis using SNPs associated with CHD as IVs. Among them, the results of the most essential IVW analysis method showed the effect of CHD on anti-H. pylori IgG seropositivity was not statistically significant (IVW: OR=0.979, 95% CI:0.290–3.302, P = 0.973; Table 2). Therefore, there was no evidence of an effect of CHD on anti-H. pylori IgG seropositivity from several different MR analysis methods.

## 4. Discussion

In recent years, the relationship between H. pylori infection and CHD has focused on the academic community. Observational studies have shown that the occurrence of CHD is closely related to anti-H. pylori IgG seropositivity. However, these studies may be affected by confounding factors and reverse causation, resulting in a causal relationship that is still difficult to identify. Genetic variants can be identified as MR analysis IVs to assess the causal relationship between exposure and outcome. Due to genetic variants being randomly assigned during the formation of human gametes, and genotypes were typically unaffected by the external environment, the MR method could largely avoid and overcome the limitations of confounding factors and reverse causality common in observational study(29, 30). In the study we found direct evidence showing the causal relationship between anti-H. pylori IgG seropositivity on CHD using an MR analysis method. Although the MR estimates using IVW, MR Egger, and weighted median analysis were inconsistent, IVW analysis supports a causal association between anti-H. pylori IgG seropositivity and CHD. The statistical power of the IVW method is significantly higher than that of other MR methods [42]. Thus, the results of this MR analysis indicated that anti-H. pylori IgG seropositivity may increase the risk of CHD in European. The reverse MR analysis found no evidence that CHD increases anti-H risk. pylori IgG seropositivity. The bidirectional study guaranteed the inference of causality in both directions. In addition, we also revealed the causal relationship between anti-H. pylori IgG seropositivity on peak insulin response, total free cholesterol levels, waist circumference, and waist-to-hip ratio. We concluded that increased anti-H. pylori IgG seropositivity is associated with increased risks of CHD in the European population, which may be explained by lower total free cholesterol levels or waist circumference or waist-to-hip ratio. Among them, lower waist circumference or waist-to-hip ratio increased anti-H. pylori IgG seropositivity is associated with increased risks of CHD in the European female population. Moreover, we also discovered that increased anti-H. pylori IgG seropositivity is associated with increased risks of CHD in the Hispanic or Latin American population, which might be explained by higher peak insulin response.

In 1994, Mendall et al. first reported the relationship between H. pylori infection and CHD, which has attracted the attention of many scholars [44]. Recent studies have confirmed that H. pylori infection is an independent risk factor for AS and CHD [45]. The mechanism of CHD caused by H. pylori infection is still unclear, and some studies believe it may be related to inflammatory response, free radical action, and autoimmunity. One prominent explanation is that H. pylori infection leads to chronic inflammation, associated with an increased risk of CHD [10]. H. pylori infection has been shown to trigger a chronic inflammatory response, and this response may lead to endothelial dysfunction and accelerated atherosclerosis. Additionally, H. pylori may affect lipid metabolism, a risk factors for CHD [46]. A cohort study showed that H. pylori infection may play a pathophysiologic role in developing dyslipidemia, whereas H. pylori eradication might decrease the risk of dyslipidemia [47]. Another pathogenesis possibility is that H. pylori infection leads to immune system activation, which can result in elevated levels of circulating immune complexes and cytokines. These immune complexes and cytokines may promote a prothrombotic state and lead to thrombus formation in the coronary arteries, ultimately causing CHD. Furthermore, H. pylori infection has been associated with increased platelet activation and aggregation, which can also contribute to thrombus formation, further leading to CHD [48]. Since most of the explanations lack supporting evidence, the mechanism between anti-H. pylori IgG seropositivity and CHD still deserve exploration.

Remarkably, our study revealed that the causal relationship between anti-H. pylori IgG seropositivity and lower total free cholesterol levels or higher peak insulin response. Previous MR studies have found that increased waist circumference or higher waist-to-hip ratio is associated with an increased risk of CHD [49,50]. However, our MR study found that lower waist circumference or waist-to-hip ratio increased anti-H. pylori IgG seropositivity is associated with increased risks of CHD in the European female population. However further research is needed to clarify the results as there is no obvious explanation for how H. pylori infection reduces waist circumference or waist-to-hip ratio. Future studies are required in order to determine the potential pathogenesis of anti-H. pylori IgG seropositivity among these four mediators.

This MR study provided strong evidence of a causal relationship between anti-H. pylori IgG seropositivity and related to an increased risk of CHD. Therefore, H. pylori infection may play a role in the development of CHD. Our study has shown that anti-H. pylori IgG seropositivity is significantly associated with an increased risk of CHD (OR=1.003, 95% CI:1.0001.006, P = 0.048) and increases in peak insulin response (β = 0.214, 95% CI: 0.025–0.403, P = 0.026) or decreases in total free cholesterol levels (β = －0.031, 95% CI: －0.061 to －0.000, P = 0.045), waist circumference (β = －0.073, 95% CI: －0.138 to －0.008, P = 0.027) and waist-to-hip ratio (β = －0.069, 95% CI: －0.136 to －0.002, P = 0.044). This study provides valuable advice for clinical early prevention of CHD. The advantages of our research include several aspects. Firstly, we examined the causal relationship between anti-H. pylori IgG seropositivity on CHD and peak insulin response, total free cholesterol levels, waist circumference, or waist-to-hip ratio using MR analysis to exclude confounders, providing strong pieces of evidence for causal relationship between H. pylori infection and CHD. Secondly, we performed various MR analysis methods and comprehensive sensitivity analyses, and heterogeneity testing to validate our research results. Moreover, we conducted a bidirectional MR analysis to avoid reverse causality and confirmed the findings of observational studies with less bias. However, our study also has two limitations. To begin, it is impossible to determine the exact time and degree of H. pylori infections in patients with anti-H. pylori IgG seropositivity, which may not be possible to determine whether it is a past infection or an ongoing infection. Therefore, false negative or false positive results may exist, potentially exaggerating the association between the H. pylori infection and CHD. Second, we used a more relaxed threshold (p < 5 × 10^−5^) when we selected IVs. While this approach may enhance statistical power, including more instrumental variables in the study increases the risk of introducing instrumental variables with multiple effects. To address potential horizontal pleiotropy, we performed sensitivity analyses, including MR-Egger intercept, MR-PRESSO, and leave-one-out analysis.

## 5. Conclusion

Our MR study provides evidence supporting a unidirectional causal association between anti-H. pylori IgG seropositivity on CHD at the genetic level. Anti-H. pylori IgG seropositivity is associated with an increased risk of CHD, which may be related to a higher peak insulin response or lower total free cholesterol levels, as well as a decrease in waist circumference and waist-to-hip ratio. Therefore, eradication H. pylori infection may be effective in the prevention of CHD. However, clinical studies are needed to confirm further whether control of H. pylori infection reduces the risk of CHD.

## Supporting information

S1 File STROBE-MR Checklist: Information Items to be Included in Mendelian Randomization Studies. (DOCX)

S1 Table Results data from this Mendelian randomization study. (CSV)
