# Supplementary material for: Association between Anti-Helicobacter pylori IgG seropositivity on coronary heart disease and potential pathogenesis: a Mendelian randomization study
Source: PLoS One. 2025 Dec 9;20(12):e0329137. doi: 10.1371/journal.pone.0329137 (PMC12688094; doi:10.1371/journal.pone.0329137)
Supplement: S1 File — (DOCX) [file pone.0329137.s001.docx]

**STROBE-MR checklist of recommended items to address in reports of Mendelian randomization studies**^1^ ^2^

| **Item No.** | **Section** | **Checklist item** | **Page No.** | **Relevant text from manuscript** |
| --- | --- | --- | --- | --- |
| 1 | **TITLE and ABSTRACT** | Indicate Mendelian randomization (MR) as the study’s design in the title and/or the abstract if that is a main purpose of the study | 1-3 | Association between Anti-Helicobacter pylori IgG seropositivity on coronary heart disease and potential pathogenesis: a Mendelian randomization study |
|  | **INTRODUCTION** |  |  |  |
| 2 | **Background** | Explain the scientific background and rationale for the reported study. What is the exposure? Is a potential causal relationship between exposure and outcome plausible? Justify why MR is a helpful method to address the study question | 2 | Currently, observational studies and clinical trials have shown that the occurrence of coronary heart disease (CHD) is closely related to anti-Helicobacter pylori (H. pylori) IgG seropositivity, but these studies may be affected by confounding factors, resulting in a causal relationship that is still controversial. |
| 3 | **Objectives** | State specific objectives clearly, including pre-specified causal hypotheses (if any). State that MR is a method that, under specific assumptions, intends to estimate causal effects | 2 | We conducted a Mendelian randomization analysis to clarify the association between anti-H. pylori IgG seropositivity and CHD and explore its potential pathogenesis. |
|  | **METHODS** |  |  |  |
| 4 | **Study design and data sources** | Present key elements of the study design early in the article. Consider including a table listing sources of data for all phases of the study. For each data source contributing to the analysis, describe the following: |  |  |
|  | a) | Setting: Describe the study design and the underlying population, if possible. Describe the setting, locations, and relevant dates, including periods of recruitment, exposure, follow-up, and data collection, when available. | 5，7 | We applied an MR design to elucidate the causal association between anti-H. pylori IgG seropositivity on CHD and mediators. An MR research design must be based on the following three core assumptions:(1) the instrumental variables (IVs) are significantly associated with anti-H. pylori IgG seropositivity; (2) the IVs should not be related to potential confounding factors that affect both exposure or outcome; (3) the IVs should exert effects on the outcome only through the exposure (Fig 1).  Table 1 |
|  | b) | Participants: Give the eligibility criteria, and the sources and methods of selection of participants. Report the sample size, and whether any power or sample size calculations were carried out prior to the main analysis | 7 | Table 1 |
|  | c) | Describe measurement, quality control and selection of genetic variants | 6 | We obtained the publicly available GWAS summary statistic. |
|  | d) | For each exposure, outcome, and other relevant variables, describe methods of assessment and diagnostic criteria for diseases | 6 | Exposure, outcome, and mediator data were obtained all from the genome-wide association studies (GWAS) summary data. |
|  | e) | Provide details of ethics committee approval and participant informed consent, if relevant | 19 | No additional ethical review was required since our study used publicly available summary data. |
| 5 | **Assumptions** | Explicitly state the three core IV assumptions for the main analysis (relevance, independence and exclusion restriction) as well assumptions for any additional or sensitivity analysis | 5 | An MR research design must be based on the following three core assumptions:(1) the instrumental variables (IVs) are significantly associated with anti-H. pylori IgG seropositivity; (2) the IVs should not be related to potential confounding factors that affect both exposure or outcome; (3) the IVs should exert effects on the outcome only through the exposure (Fig 1). |
| 6 | **Statistical methods: main analysis** | Describe statistical methods and statistics used |  |  |
|  | a) | Describe how quantitative variables were handled in the analyses (i.e., scale, units, model) | 8 | MR analysis of genetic variation must be related to exposure but not affected by confounders. |
|  | b) | Describe how genetic variants were handled in the analyses and, if applicable, how their weights were selected | 8 | Firstly, the SNPs related to the exposure and outcome were standardized to ensure alignment of allelic directions and removal of palindromic sequences. Then, SNPs were excluded if unavailable in outcome datasets or defined as ambiguous palindromic SNPs with minor allele frequencies >0.42 and <0.58. In this study, three SNPs were eliminated. Moreover, before we conducted MR analysis, we used the MR-PRESSO method to detect outliers to enhance the robustness of the results. |
|  | c) | Describe the MR estimator (e.g. two-stage least squares, Wald ratio) and related statistics. Detail the included covariates and, in case of two-sample MR, whether the same covariate set was used for adjustment in the two samples | 9 | We primarily adopted the IVW method under the fixed-effect model in this MR study. In addition, multiple complementary analyses were conducted to ensure the results' stability and reliability, such as IVW under the random-effects model, weighted median, and MR-Egger. |
|  | d) | Explain how missing data were addressed |  |  |
|  | e) | If applicable, indicate how multiple testing was addressed | 9 | To validate the dependability of the primary findings, conducting sensitivity analysis has been pivotal in uncovering potential pleiotropy and heterogeneity within MR estimates. |
| 7 | **Assessment of assumptions** | Describe any methods or prior knowledge used to assess the assumptions or justify their validity | 9 | The statistical power of the inverse-variance weighted (IVW) method is significantly higher than that of other MR methods. |
| 8 | **Sensitivity analyses and additional analyses** | Describe any sensitivity analyses or additional analyses performed (e.g. comparison of effect estimates from different approaches, independent replication, bias analytic techniques, validation of instruments, simulations) | 9 | To determine whether a single SNP drives the causal signal, we also conducted a Leave-one-out analysis. |
| 9 | **Software and pre-registration** |  |  |  |
|  | a) | Name statistical software and package(s), including version and settings used | 10 | A total of MR analyses was performed using the RStudio package“TwosampleMR”. |
|  | b) | State whether the study protocol and details were pre-registered (as well as when and where) |  |  |
|  | **RESULTS** |  |  |  |
| 10 | **Descriptive data** |  |  |  |
|  | a) | Report the numbers of individuals at each stage of included studies and reasons for exclusion. Consider use of a flow diagram | 10 | Before we performed MR estimation, no outliers with large pleiotropy were detected by MR-PRESSO analysis in this study. In this study, three ambiguous palindromic SNPs (rs7281117, rs761648, rs8051818) defined as having minor allele frequencies were eliminated. |
|  | b) | Report summary statistics for phenotypic exposure(s), outcome(s), and other relevant variables (e.g. means, SDs, proportions) | 11 | Table 2 |
|  | c) | If the data sources include meta-analyses of previous studies, provide the assessments of heterogeneity across these studies |  |  |
|  | d) | For two-sample MR:  i.  Provide justification of the similarity of the genetic variant-exposure associations between the exposure and outcome samples  ii.  Provide information on the number of individuals who overlap between the exposure and outcome studies | 12 | We performed MR-PRESSO analysis to identify outliers exhibiting pleiotropy and conducted MR estimates. |
| 11 | **Main results** |  |  |  |
|  | a) | Report the associations between genetic variant and exposure, and between genetic variant and outcome, preferably on an interpretable scale | 11 | Table 2 |
|  | b) | Report MR estimates of the relationship between exposure and outcome, and the measures of uncertainty from the MR analysis, on an interpretable scale, such as odds ratio or relative risk per SD difference | 11 | Table 2 |
|  | c) | If relevant, consider translating estimates of relative risk into absolute risk for a meaningful time period | 11 | Table 2 |
|  | d) | Consider plots to visualize results (e.g. forest plot, scatterplot of associations between genetic variants and outcome versus between genetic variants and exposure) | 11,13 | Fig 2-5 |
| 12 | **Assessment of assumptions** |  |  |  |
|  | a) | Report the assessment of the validity of the assumptions | 10 | In this MR analysis, the IVW method analysis under fixed-effect showed evidence to support a causal association between anti-H. pylori IgG seropositivity and CHD (OR=1.003 95% CI:1.000-1.006, P=0.048). Under a random-effects model, we obtained similar risk results (OR=1.003, 95% CI:1.000-1.005, P=0.026). |
|  | b) | Report any additional statistics (e.g., assessments of heterogeneity across genetic variants, such as *I^2^*, Q statistic or E-value) | 11 | To test the heterogeneity of the study, Cochran's Q test indicated no evidence of heterogeneity between IV estimates based on the individual variants (MR Egger: P=0.900, IVW: P=0.912; Fig 2C). |
| 13 | **Sensitivity analyses and additional analyses** |  |  |  |
|  | a) | Report any sensitivity analyses to assess the robustness of the main results to violations of the assumptions | 11 | Table 2 |
|  | b) | Report results from other sensitivity analyses or additional analyses | 11 | Table 2 |
|  | c) | Report any assessment of direction of causal relationship (e.g., bidirectional MR) | 14 | There was no evidence of an effect of CHD on anti-H. pylori IgG seropositivity from several different MR analysis methods. |
|  | d) | When relevant, report and compare with estimates from non-MR analyses |  |  |
|  | e) | Consider additional plots to visualize results (e.g., leave-one-out analyses) | 11 | Results from the leave-one-out analysis demonstrated that no single SNP was driving the IVW point estimate (Fig 2D). |
|  | **DISCUSSION** |  |  |  |
| 14 | **Key results** | Summarize key results with reference to study objectives | 14,15 | In the study we found direct evidence showing the causal relationship between anti-H. pylori IgG seropositivity on CHD using an MR analysis method.  In addition, We concluded that increased anti-H. pylori IgG seropositivity is associated with increased risks of CHD in the European population, which may be explained by lower total free cholesterol levels or waist circumference or waist-to-hip ratio. Among them, lower waist circumference or waist-to-hip ratio increased anti-H. pylori IgG seropositivity is associated with increased risks of CHD in the European female population. Moreover, we also discovered that increased anti-H. pylori IgG seropositivity is associated with increased risks of CHD in the Hispanic or Latin American population, which might be explained by higher peak insulin response. |
| 15 | **Limitations** | Discuss limitations of the study, taking into account the validity of the IV assumptions, other sources of potential bias, and imprecision. Discuss both direction and magnitude of any potential bias and any efforts to address them | 17 | However, our study also has two limitations. To begin, it is impossible to determine the exact time and degree of H. pylori infections in patients with anti-H. pylori IgG seropositivity, which may not be possible to determine whether it is a past infection or an ongoing infection. Therefore, false negative or false positive results may exist, potentially exaggerating the association between the H. pylori infection and CHD. Second, we used a more relaxed threshold (p < 5 × 10−5) when we selected IVs. While this approach may enhance statistical power, including more instrumental variables in the study increases the risk of introducing instrumental variables with multiple effects. |
| 16 | **Interpretation** |  |  |  |
|  | a) | Meaning: Give a cautious overall interpretation of results in the context of their limitations and in comparison with other studies | 18 | However, clinical studies are needed to confirm further whether control of H. pylori infection reduces the risk of CHD. |
|  | b) | Mechanism: Discuss underlying biological mechanisms that could drive a potential causal relationship between the investigated exposure and the outcome, and whether the gene-environment equivalence assumption is reasonable. Use causal language carefully, clarifying that IV estimates may provide causal effects only under certain assumptions | 15 | We concluded that increased anti-H. pylori IgG seropositivity is associated with increased risks of CHD in the European population, which may be explained by lower total free cholesterol levels or waist circumference or waist-to-hip ratio. Among them, lower waist circumference or waist-to-hip ratio increased anti-H. pylori IgG seropositivity is associated with increased risks of CHD in the European female population. Moreover, we also discovered that increased anti-H. pylori IgG seropositivity is associated with increased risks of CHD in the Hispanic or Latin American population, which might be explained by higher peak insulin response. |
|  | c) | Clinical relevance: Discuss whether the results have clinical or public policy relevance, and to what extent they inform effect sizes of possible interventions | 18 | Therefore, eradication H. pylori infection may be effective in the prevention of CHD. |
| 17 | **Generalizability** | Discuss the generalizability of the study results (a) to other populations, (b) across other exposure periods/timings, and (c) across other levels of exposure |  |  |
|  | **OTHER INFORMATION** |  |  |  |
| 18 | **Funding** | Describe sources of funding and the role of funders in the present study and, if applicable, sources of funding for the databases and original study or studies on which the present study is based | 19 | This study was financially supported by the Health Science and Technology Fund Project of Jilin Provincial Health Commission (2023JC044); Chinese Medicine Evidence Based Capacity Enhancement Program (2024) of the Affiliated Hospital to Changchun University of Chinese Medicine. |
| 19 | **Data and data sharing** | Provide the data used to perform all analyses or report where and how the data can be accessed, and reference these sources in the article. Provide the statistical code needed to reproduce the results in the article, or report whether the code is publicly accessible and if so, where | 18 | The original contributions presented in the study inquiries can be directed to the corresponding authors. |
| 20 | **Conflicts of Interest** | All authors should declare all potential conflicts of interest | 18 | The authors declare no competing interests. |

This checklist is copyrighted by the Equator Network under the Creative Commons Attribution 3.0 Unported (CC BY 3.0) license.

1. Skrivankova VW, Richmond RC, Woolf BAR, Yarmolinsky J, Davies NM, Swanson SA, et al. Strengthening the Reporting of Observational Studies in Epidemiology using Mendelian Randomization (STROBE-MR) Statement. JAMA. 2021;under review.

2. Skrivankova VW, Richmond RC, Woolf BAR, Davies NM, Swanson SA, VanderWeele TJ, et al. Strengthening the Reporting of Observational Studies in Epidemiology using Mendelian Randomisation (STROBE-MR): Explanation and Elaboration. BMJ. 2021;375:n2233.
